# T wave abnormalities, high body mass index, current smoking and high lipoprotein (a) levels predict the development of major abnormal Q/QS patterns 20 years later. A population-based study

**DOI:** 10.1186/1471-2261-6-10

**Published:** 2006-03-06

**Authors:** Christina Strom Moller, Liisa Byberg, Johan Sundstrom, Lars Lind

**Affiliations:** 1 Department of Public Health and Caring Sciences/Geriatrics, Uppsala University, Sweden; 2 Department of Medical Science, Uppsala University, Sweden; 3 AstraZeneca, Research and Development, Sweden

## Abstract

**Background:**

Most studies on risk factors for development of coronary heart disease (CHD) have been based on the clinical outcome of CHD. Our aim was to identify factors that could predict the development of ECG markers of CHD, such as abnormal Q/QS patterns, ST segment depression and T wave abnormalities, in 70-year-old men, irrespective of clinical outcome.

**Methods:**

Predictors for development of different ECG abnormalities were identified in a population-based study using stepwise logistic regression. Anthropometrical and metabolic factors, ECG abnormalities and vital signs from a health survey of men at age 50 were related to ECG abnormalities identified in the same cohort 20 years later.

**Results:**

At the age of 70, 9% had developed a major abnormal Q/QS pattern, but 63% of these subjects had not been previously hospitalized due to MI, while 57% with symptomatic MI between age 50 and 70 had no major Q/QS pattern at age 70. T wave abnormalities (Odds ratio 3.11, 95% CI 1.18–8.17), high lipoprotein (a) levels, high body mass index (BMI) and smoking were identified as significant independent predictors for the development of abnormal major Q/QS patterns. T wave abnormalities and high fasting glucose levels were significant independent predictors for the development of ST segment depression without abnormal Q/QS pattern.

**Conclusion:**

T wave abnormalities on resting ECG should be given special attention and correlated with clinical information. Risk factors for major Q/QS patterns need not be the same as traditional risk factors for clinically recognized CHD. High lipoprotein (a) levels may be a stronger risk factor for silent myocardial infarction (MI) compared to clinically recognized MI.

## Background

Several longitudinal studies have investigated the impact of different ECG abnormalities on future development of coronary heart disease (CHD) with morbidity, based on episodes of chest pain or mortality as end-points [1], excluding those with silent myocardial infarction (MI). The prevalence and prognosis of silent MI is still controversial [2,3] and it remains uncertain whether patients with silent MI have unique characteristics that distinguish them from those with recognized events [4]. In the present population-based study with 20 years of follow-up, our primary objective was to identify predictors for the development of abnormal major Q/QS patterns as a marker for MI, irrespective of chest pain, in order to obtain a complementary picture to previous studies. Our secondary objective was to investigate predictors for ST segment depression and T wave abnormalities. Among the possible predictors were anthropometrical and metabolic factors, as well as ECG items and vital signs.

## Methods

### Study sample

The Uppsala Study of Adult Men (ULSAM) is a longitudinal study based on all available 50-year-old men in Uppsala, Sweden, born between 1920 and 1924. They were invited to a health survey aimed at identifying risk factors for cardiovascular disease [5]. Of the invited subjects, 2,322 (82%) participated in 1970–1973. In 1991–1995, eligible participants were invited for re-examination at age 70. Of these, 1,221 (73%) participated. Between the 2 surveys, 219 had moved and 422 had died 128 due to acute MI. All subjects gave informed consent, and the Ethics Committee of Uppsala University approved the study. Official hospital discharge register data was available.

In the present study, all subjects hospitalized due to acute MI before the 50-year survey (n = 8) or with major Q wave (n = 6) or left bundle branch block (LBBB) (n = 5) on ECG at age 50, were excluded from further analyses, resulting in a study population of 2303 men at age 50 of which 1131 men were re-investigated at age 70. Development of ST segment depression and isolated T wave abnormality on ECG at age 70 were investigated in subgroups of 1006 men (additional exclusion of men with Minnesota codes 4.1–4.2 at age 50) and 858 men (additional exclusion of men with Minnesota codes 4.1–4.2 at age 50 and/or 70, and 5.1–5.4 at age 50), respectively.

### Investigations and laboratory methods

#### Investigations at age 50

These investigations have been described extensively elsewhere [5]. 12-lead resting ECGs were recorded, including standard leads I, II and III, unipolar leads aVR, aVL and aVF and V1-6. Two experienced physicians at the Department of Clinical Physiology classified the ECGs according to the Minnesota classification system [6,7] (Table 1). The Minnesota codes were assessed on 12-lead ECGs. ST depression was not tabulated when found in less than a majority of beats of the leads and when the baseline was swinging widely. Flat or inverted T waves were accepted in leads III, aVL and V1. Body mass index (BMI) was calculated as weight (in kg) divided by height (in meters) squared. Blood pressure (BP) was measured in the recumbent position after a 10 minutes rest with a mercury manometer (Kifa Ercameter, wall-model). Blood glucose was measured by spectrophotometry using the glucose oxidase method. The serum insulin was determined with the Phadebas Insulin Test (Pharmacia AB, Uppsala, Sweden). Concentrations of serum low-density lipoprotein (LDL)-cholesterol, high-density lipoproteine (HDL)-cholesterol and apolipoprotein AI (apoA1) and apolipoprotein B (apoB) were performed on serum samples stored in liquid nitrogen since sampling. ApoB were determined by a two-site immunoradiometric assay and apoA1 by a competitive radioimmunoassay. Coding of smoking habits was based on interview reports.

**Table 1:** Minnesota classification of ECGs

ECG abnormalities	Minnesota Code	Definition
Q or QS pattern	1.1	Q duration ≥ 0.04s in I, II, V2–V6.
		Q duration ≥ 0.05s in both aVF and III.
		QS pattern through V1–V4, V5 and V6.
		QS pattern when R wave is present in adjacent precordial lead to the right V2–V6.
ST segment depression	4.1–4.2	4.1: ST-depression ≥ 1 mm in I, II, aVL, aVF, V1–V6.
		4.2: ST depression 0.5–0.9 mm in I, II, aVL, aVF, V1–V6.
T wave items	5.1–5.4	5.1: T amplitude ≥ 5 mm in I, II V2–V6 when R amplitude ≥ 5 mm in aVL when QRS mainly upright in aVF.
		5.2: T amplitude = -1 to -5 in I, II, V2–V6 when R amplitude ≥ 5 mm in aVL when QRS mainly upright in aVF.
		5.3: T wave flat or small diphasic in I, II, V2–V6 when R amplitude ≥ in aVL when QRS mainly upright in aVF.
		5.4: T amplitude positive and T/R amplitude radio <1/20 in any of leads I, aVL, V6: R wave amplitude must be ≥ 10.0 mm.

#### Investigations at age 70

The cohort was reinvestigated 20 years later and a standard resting ECG was obtained and classified according to the Minnesota code [6,7] by one experienced physician.

### ECG abnormalities

Our primary outcome, a finding of a major abnormal Q/QS pattern was defined according to the Minnesota code 1.1 [6,7]. Minor abnormal Q/QS patterns that only fulfilled the requirements of Minnesota code 1.2 or 1.3 were not included as end-points reducing the possibility of inclusion of abnormal Q/QS patterns of other aetiologies than CHD. Our secondary end-points were ST segment depression (Minnesota 4.1–4.2) and T wave abnormalities (Minnesota 5.1–5.4).

Subjects with ECG abnormalities observed during the survey were not further assessed by a cardiologist or evaluated further by other cardiac examinations as part of the protocol.

### Statistical analyses

The distribution of a continuous variable was tested for normality using Shapiro Wilk's test. Skewed variables were logarithmically transformed to reach normal distribution. Univariate regression analyes were performed on the list of candidate predictor variables at age 50, comparing those with and without ECG abnormalities at age 70 using χ^2 ^test or ANOVA. A p < 0.05 was considered statistically significant. All candidate predictors with a univariate p < 0.15 were evaluated for independent association in a backward stepwise logistic regression model. Continuous variables were standardized to one standard deviation (SD) to determine the magnitude of the relationship to, and the statistical significance of the predictors of each of the defined outcomes in the regression analyses. The robustness of the final model was checked by including previously removed variables. As an additional analysis, the same variables were tested in the same model but substituting the end point major Q/QS pattern by death due to acute MI before the 70-year examination. The prevalence of T wave abnormalities at age 50 in the subjects who died during the 20-year follow-up period was also compared to those who survived using χ^2 ^test.

## Results

The prevalence of CHD disease-related ECG items in ULSAM at ages 50 and 70 are shown in Table 2. In our cohort, 9% (n = 102) developed a major abnormal Q/QS pattern by the age of 70 but 63% (n = 64) of these subjects had not been hospitalized due to MI. Among the 89 men that had been hospitalized due to MI at any time-point during the 20 years follow-up, 57% (n = 51) had no major Q/QS pattern on ECG at the age of 70. The prevalence of T wave abnormalities had increased more than three-fold and the prevalence of ST segment depression had increased more than six-fold.

**Table 2:** Prevalence of ECG abnormalities at age 50 and 70

ECG abnormalities *	**Age 50 (n = 2314) n (%)**	**Age 70 (n = 1135) n (%)**
Major Q or QS pattern (1.1)	6 (0.26)	102 (9.0)
Minor Q or QS pattern (1.2/1.3)	20 (0.86)	49 (4.32)
ST seg. depression (4.1/4.2)	50 (2.16)	161 (14.19)
T wave items (5.1–5.4)	130 (5.62)	180 (15.86)
LBBB (7.1)	5 (0.22)	18 (1.59)
High R-amplitude (3.1–3.3)	336 (14.52)	269 (26.8)
Atrial fibrillation/flutter (8.3)	7 (0.30)	55 (4.85)

* Minnesota codes in parenthesis.

LBBB, left bundle branch block

No attempt was made to eliminate overlap due to the occurrence of several items in one ECG. Subjects with acute MI before first survey were excluded from the analysis.

### Major abnormal Q/QS pattern

When subjects with a new major abnormal Q/QS pattern at age 70 (n = 102) were compared with those who had not developed such ECG changes during the follow-up period (n = 1033), those with abnormal Q/QS pattern had elevated levels of BMI, serum triglycerides, serum cholesterol, Lp (a) and apoB at age 50 (Table 3). There was no statistically significant difference between the groups regarding some of the traditional risk factors for CHD including smoking and SBP. There was a higher prevalence of T wave abnormalities and minor Q/QS waves in the group with major abnormal Q/QS pattern (Table 4). Independent risk factors for development of a new major abnormal Q/QS pattern on the ECG at age 70 were high Lp (a), high BMI, current smoking and T wave abnormalities all contributing to an increased risk (Table 5). There was more than a three-fold risk for men with a T wave abnormality at age 50 to develop a new major abnormal Q/QS pattern at age 70 compared to men without T wave abnormalities.

**Table 3:** Clinical characteristics at age 50 according to Q-QS pattern at age 70 (mean ± SD)

Variables, age 50	Abnormal Q-QS pattern (1.1) at age 70	n	No abnormal Q-QS (1.1) pattern at age 70	n	p value
BMI (kg/m2)	25.6 (3.2)	101	24.7 (2.9)	1030	0.003
SBP supine (mmHg)	134.4 (16.8)	101	131.3 (16.7)	1030	0.08
DBP supine (mmHg)	84.3 (11.4)	101	82.5 (10.4)	1030	0.1
Fasting B-gluc (mmol/l)	5.1 (1.1)	101	4.9 (0.6)	1029	0.2
Fasting insulin (μU/ml)	12.4 (7.9)	73	12.3 (6.5)	850	0.9
Serum TG (mmol/l)	2.0 (1.0)	101	1.8(0.9)	1030	0.04
Serum CHOL (mmol/l)	7.0 (1.1)	101	6.8 (1.3)	1030	0.06
HDL CHOL (mmol/l)	1.3 (0.3)	72	1.4 (0.4)	844	0.2
LDL CHOL (mmol/l)	5.4 (1.2)	72	5.2 (1.2)	844	0.2
Lp (a) (U/l)	322.2 (385.6)	72	234.8 (295.8)	815	0.01
ApoA1 (g/l)	1.4 (0.2)	76	1.4 (0.2)	817	0.7
ApoB (g/l)	1.3 (0.2)	76	1.2 (0.3)	817	0.03
ApoB/ApoA1 ratio	0.91 (0.2)	76	0.86 (0.2)	817	0.1
Smoking (%)	53 (52.5)	101	446 (43.3)	1030	0.08

BMI, Body Mass Index; HR, heart rate; SBP, systolic blood pressure; DBP, diastolic blood pressure; CHOL, cholesterol; HDL, high-density lipoproteins; LDL, low-density lipoproteins; Lp(a), lipoprotein (a); ApoA1, apolipoprotein A1; ApoB, apolipoprotein B.

**Table 4:** ECG findings at age 50 according to Q-QS-pattern at age 70

ECG abnormalities* at age 50	Abnormal Q-QS pattern (1.1) at age 70 (n = 102) n (%)		No abnormal Q-QS pattern (1.1) at age 70 (n = 1033) n (%)	p value
Minor Q-QS pattern (1.2/1.3)	2 (2.0)		4 (0.4)	0.04
High amplitude R-wave (3.1/3.3)	11 (10.9)		148 (14.4)	0.3
ST-segment depression (4.1/4.2)	1 (1.0)		11 (1.0)	0.9
T-wave items (5.1–5.4)	8 (7.9)		31 (3.0)	0.01
AF/flutter (8.3)	0 (0)		3 (0.3)	0.6

* Minnesota codes in parenthesis

AF, atrial fibrillation

**Table 5:** Multivariate logistic regression analysis for development of abnormal Q-QS pattern (Minnesota 1.1) on ECG at age 70

Variable	Odds Ratio (95% - CI)	p value
BMI (1SD = 3.3 kg/m2)	1.32 (1.03–1.68)	0.03
Lp (a)	1.35 (1.05–1.73)	0.02
T-wave abnormality*	3.11 (1.18–8.17)	0.02
Smoking	1.63 (1.01–2.65)	0.046

Odds ratios from logistic regression were applied to variables standardised to one standard deviation and adjusted for age at entry.

*Minnesota code 5.1–5.4

### ST segment depression

Of the 1006 men free of ST segment depression at age 50, 13% had developed ST segment depression at the age of 70. In multiple logistic regression analysis T wave abnormalities (OR = 3.94, 95% CI 1.58–9.82, p = 0.003) and high fasting blood glucose levels (OR = 1.25, 95% CI 1.02–1.54, p = 0.035) were independent predictor for ST segment depression.

### Isolated T wave abnormality

Of the 858 men without ST segment depression (at age 50 and/or 70) and T wave abnormality at age 50, 9% had developed an isolated T wave abnormality at the age of 70. An increase in BMI by 1SD increased the risk of having a T wave abnormality at the age of 70 by 50% (OR = 1.48, 95% CI 1.14–1.92). None of the other variables tested predicted future development of a T wave abnormality. Subjects who died due to MI during the observation period also had a significantly higher prevalence of T-wave abnormalities at age 50 compared to those who survived (15.3% vs 4.8%, p < 0.01).

### Death due acute MI

Independent risk factors at age 50 for death due to acute MI before the 70-year examination were high BMI, high DBP, high ApoB/ApoA1 ratio, T wave abnormalities, smoking and diabetes mellitus (Table 6).

**Table 6:** Multivariate logistic regression analysis for death due to myocardial infarction before 70-year examination

Variable	Odds Ratio (95% - CI)	p value
BMI (1SD = 3.3 kg/m2)	1.29 (1.01–1.57)	0.009
DBP supine (mmHg)	1.66 (1.37–2.02)	<0.001
ApoB/ApoA1 ratio	1.50 (1.23–1.83)	<0.001
T-wave abnormality*	2.66 (1.45–4.89)	0.002
Smoking	1.81 (1.17–2.82)	0.008
Diabetes mellitus	2.05 (1.03–4.07)	0.03

Odds ratios from logistic regression were applied to variables standardised to one standard deviation and adjusted for age at entry.

*Minnesota code 5.1–5.4

## Discussion

Besides the presence of T wave abnormalities, we identified high BMI, high Lp (a) and smoking at age 50 as independent risk factors for the development of major abnormal Q/QS patterns at age 70. T wave abnormalities, together with high fasting blood glucose were also important predictors for the development of ST segment depression while participants with higher BMI had an increased risk of developing T wave abnormalities.

This is a longitudinal community based study, with long follow-up period, where all subjects from one birth cohort in the community were invited to participate. The same two nurses registered the ECGs at both surveys, contributing to consistency in electrode placement. Three physicians carried out the coding of all ECGs, whereby the coding consistency can be expected to be high. However, coding errors may confound analysis of ECG data. An obvious limitation of our study is that women were not included. There was no validation of the outcome detection system, however, the myocardial infarction diagnosis in the Swedish hospital register has been found to be correct in 93–97% of cases with less than one percent being missed [8].

Our study is unique in that it uses ECG variables as outcomes instead of clinical outcomes. By choosing major abnormal Q/QS patterns as end-point, silent myocardial infarctions have also been included in the analyses. According to most studies a clinically unrecognized MI is believed to be associated with the same risk as recognized symptomatic MI [1,4,9] although a lower risk for unrecognized MI has also been described [10]. The prevalence of silent MI increases with age [1,2] and varies from 21% to 68% [2], with an average around 30% [11]. In our study, 63% of the subjects with a major abnormal Q/QS pattern on ECG at the 70-year survey had not been previously hospitalized due to MI. While the majority of major Q waves are believed to be due to myocardial infarction, some may be due to other causes, including myocarditis, cardiac amyloidosis, and cardiomyopathy [12]. Also displacement of the diaphragm and the heart, as in chronic obstructive lung disease may cause the appearance of a non-infarction Q wave and non-infarction Q waves may be seen in subjects with pre-excitation syndrome and left ventricular hypertrophy (LVH) [12]. The low specificity of Q/QS pattern to recognize silent myocardial necrosis is an additional limitation to our study.

Since some of the conventional risk factors [13] were not strong predictors of the development of major abnormal Q/QS patterns, our study suggests that predictors of major abnormal Q/QS patterns may be different from those predicting symptomatic MI. This is further strengthened by our additional analysis where the major Q/QS pattern end-point was substituted by death due to acute MI before the 70-year examination and where conventional risk factors such as diabetes mellitus and DBP became significant predictors. Other authors have debated whether the predisposing factors for silent MI are simply the traditional risk factors for coronary atherosclerosis, or whether silent MI patients have unique characteristics [4]. However, no general conclusions can be drawn from our study since our major abnormal Q/QS finding will include silent MI, clinically recognized MI and some non-infarction Q waves.

Loss of Q wave could explain some of the cases where diagnosis of MI was not accompanied by a visible major Q/QS pattern at age 70. Even though development of Q waves after acute MI has been considered an indicator of myocardial necrosis, studies have shown that Q wave loss may occur in 11–20% of patients after transmural MI [14,15]. The clinical significance of such an ECG phenomenon has not been fully clarified [16]. Functional recovery of stunned and/or hibernating myocardium [16], as well as regeneration of myocytes [17] has been proposed as possible mechanisms.

The pathophysiologic basis of ST segment depression on resting ECG remains speculative, and ST segment depression is seen both in left ventricular hypertrophy and myocardial ischemia [1]. One of the possible etiologies for development of left ventricular hypertrophy is altered glucose metabolism with insulin resistance and hyperinsulinemia [18,19]. On the other hand, patients with diabetes mellitus are known to have an increased risk of developing arteriosclerosis [20], so an impaired glucose metabolism could well be a predictor for ST segment depression due to either left ventricular hypertrophy or coronary heart disease.

While permanent negative T waves in the chronic stage of MI have indicated the presence of transmural infarction [21]. T waves in a post-myocardial phase have been shown to be associated with the presence of viable myocardium at jeopardy [22]. T waves should therefore be regarded as a dynamic substrate. Even though subjects not surviving the MI were not included in our main analyses, we found that the prevalence of major T wave abnormalities in those who died during the period due to acute MI was statistically significantly higher for this group compared to those who survived, suggesting that the predictive power of T wave abnormalities is not restricted to survivors of a Q wave MI.

High BMI predicted both major abnormal Q/QS pattern and abnormal T wave. One possible mechanism is horizontal displacement of the heart [23], but left ventricular hypertrophy may also play a role.

Lp (a) is believed to have potential pathogenic effects on atherosclerosis and thrombosis [24-26]. Some previous studies have identified elevated Lp (a) as a risk factor leading to premature MI [27,28], while others have shown no association between Lp (a) and risk for CHD [24]. One study has, in diabetic patients, specifically found Lp(a) to be associated with silent CHD [29]. Our study supports the finding that Lp (a) may be important an important predictor of CHD and silent MI.

The present approach using ECG-based diagnosis should be regarded as a complement to the more traditional approach used in most previous studies. One should therefore be careful not to generalize the conclusion of this study to other population groups, and further studies for confirmation of our findings are motivated.

## Conclusion

T wave abnormalities on ECG at age 50, was an independent risk factor for the development of major Q/QS abnormalities and ST segment depression on ECG at age 70. A finding of a T wave abnormality on ECG should be given special attention even though an isolated T wave change should be interpreted with caution and correlated with all available clinical and laboratory information. Risk factors for major abnormal Q/QS patterns may not be the same as risk factors for clinically recognized CHD and high Lp (a) levels may be a stronger risk factor for silent myocardial infarction (MI) compared to clinically recognized MI. Re-measurement of ECG after MI may give information on viability of myocardium through Q wave regression, which may be more frequent than previously believed.

## Competing interests

The author(s) declare that they have no competing interests.

## Authors' contributions

CSM and LB participated in the design of the study and performed the statistical analyses. CSM, LB and LL drafted the manuscript.

All authors participated in the design of the study, read and approved the final manuscript.

## Pre-publication history

The pre-publication history for this paper can be accessed here:


